# Deep Learning-Based 3D and 2D Approaches for Skeletal Muscle Segmentation on Low-Dose CT Images

**DOI:** 10.1007/s10278-025-01646-9

**Published:** 2025-08-27

**Authors:** Giuseppe Timpano, Pierangelo Veltri, Patrizia Vizza, Giuseppe Lucio Cascini, Francesco Manti

**Affiliations:** 1 Department of Surgical and Medical Sciences, Magna Graecia University, Catanzaro, 88100 Italy; 2 DIMES, University of Calabria, Rende, 87036 Italy

**Keywords:** Skeletal muscle, Deep learning, LDCT Segmentation, UNet, DeepLab

## Abstract

Automated segmentation of skeletal muscle from computed tomography (CT) images is essential for large-scale quantitative body composition analysis. However, manual segmentation is time-consuming and impractical for routine or high-throughput use. This study presents a systematic comparison of two-dimensional (2D) and three-dimensional (3D) deep learning architectures for segmenting skeletal muscle at the anatomically standardized level of the third lumbar vertebra (L3) in low-dose computed tomography (LDCT) scans. We implemented and evaluated the DeepLabv3+ (2D) and UNet3+ (3D) architectures on a curated dataset of 537 LDCT scans, applying preprocessing protocols, L3 slice selection, and region of interest extraction. The model performance was evaluated using a comprehensive set of evaluation metrics, including Dice similarity coefficient (DSC) and 95th percentile Hausdorff distance (HD95). DeepLabv3+ achieved the highest segmentation accuracy (DSC = 0.982 ± 0.010, HD95 = 1.04 ± 0.46 mm), while UNet3+ showed competitive performance (DSC = 0.967 ± 0.013, HD95 = 1.27 ± 0.58 mm) with 26 times fewer parameters (1.27 million vs. 33.6 million) and lower inference time. Both models exceeded or matched results reported in the recent CT-based muscle segmentation literature. This work offers practical insights into architecture selection for automated LDCT-based muscle segmentation workflows, with a focus on the L3 vertebral level, which remains the gold standard in muscle quantification protocols.

## Introduction

In recent years, diagnostic imaging has become increasingly central to clinical workflows, enabling both qualitative interpretation and quantitative analysis for patient assessment. Structured data extraction from radiological images improves diagnostic accuracy and supports therapeutic decision-making [1].

A key application is the quantitative assessment of skeletal muscle mass, used in clinical practice for the assessment of body composition, which, for example, is a key factor in the diagnosis of sarcopenia, a syndrome of progressive loss of muscle mass associated with reduced mobility, increased morbidity, and worse clinical outcomes. The prevalence of sarcopenia is estimated to be between 10 and 16% in individuals over the age of 60 [2–4].

Computed tomography (CT) is considered the gold standard imaging modality for muscle quantification due to its ability to differentiate soft tissues based on Hounsfield units (HU) values [5]. Among the different possible anatomical zones, the third lumbar vertebra (L3) is the most widely adopted as a standardized site for muscle measurement [6, 7].

Recently, low-dose CT (LDCT) has found increasing use in clinical screening and follow-up protocols due to its ability to minimize radiation exposure. However, lower radiation levels inherently reduce image contrast and increase noise, making soft tissue segmentation more difficult [8]. Although LDCT has been validated for clinical use in body composition analysis [9], its impact on the performance of automated segmentation algorithms remains insufficiently explored.

Manual segmentation, although still considered the gold standard, is time-consuming and subject to operator variability [10]. In this context, deep learning-based segmentation methods have been widely proposed to enable faster and more standardized analyses [11, 12]. However, despite the growing body of work on muscle segmentation, there remains a lack of systematic evaluation of the comparative performance of two-dimensional (2D) and three-dimensional (3D) deep learning models, when applied to site-specific muscle regions such as L3, both in CT and, especially, in LDCT images.

This study fills this gap by testing and comparing two segmentation architectures, (i) DeepLabv3+ (2D) and UNet3+ (3D), for automatic segmentation of skeletal muscle at the L3 vertebral level on LDCT scans, and (ii) evaluating the performance of each model in terms of segmentation accuracy, spatial coherence and computational complexity, comparing the proposed models also with the existing literature in terms of performance for muscle mass segmentation from CT images.

### Background

Automated skeletal muscle segmentation has been investigated using both slice-based two-dimensional (2D) and volumetric three-dimensional (3D) deep learning approaches. 2D models are commonly used due to their computational efficiency and compatibility with widely available axial annotations [13, 14]. However, these models do not exploit inter-slice anatomical continuity, which can lead to inconsistencies in volumetric analyses.

In contrast, 3D networks can incorporate spatial context across adjacent slices, improving segmentation coherence. Kamiya et al. [15] demonstrated the potential of 3D UNet architectures to capture surface muscle volumes from whole-body CT, while Lee et al. [16] showed advantages of volumetric models for body composition analysis on PET-CT. Nevertheless, such studies were performed on standard-dose CT and did not focus on site-specific muscle segmentation at L3.

Recent studies have introduced context-aware strategies for site-specific segmentation. Kawamoto et al. [17] and Ashino et al. [18] proposed multi-label or joint learning approaches to improve segmentation accuracy in small or complex muscle groups. However, both used layered 2D UNet architectures and did not explore direct comparisons with volumetric models.

To date, there is limited evidence on how 2D and 3D models perform when applied to the same anatomical target on LDCT images. Moreover, advanced architectures such as DeepLabv3+ and UNet3+, which integrate multi-scale features and enhanced skip connections, have not been validated for this specific application. This study aims to fill this methodological gap.

## Material and Methods

In this study, we implement and evaluate two encoder–decoder architectures for the automatic segmentation of skeletal muscle at the third lumbar vertebra (L3) level. The first architecture UNet3+ represents an evolution of the traditional UNet architecture [19], and it has been adapted to specifically address the problem of three-dimensional muscle volume segmentation. The second architecture DeepLabv3+ has instead been selected for the segmentation of muscle structures within single CT slices.

**Fig. 1:**
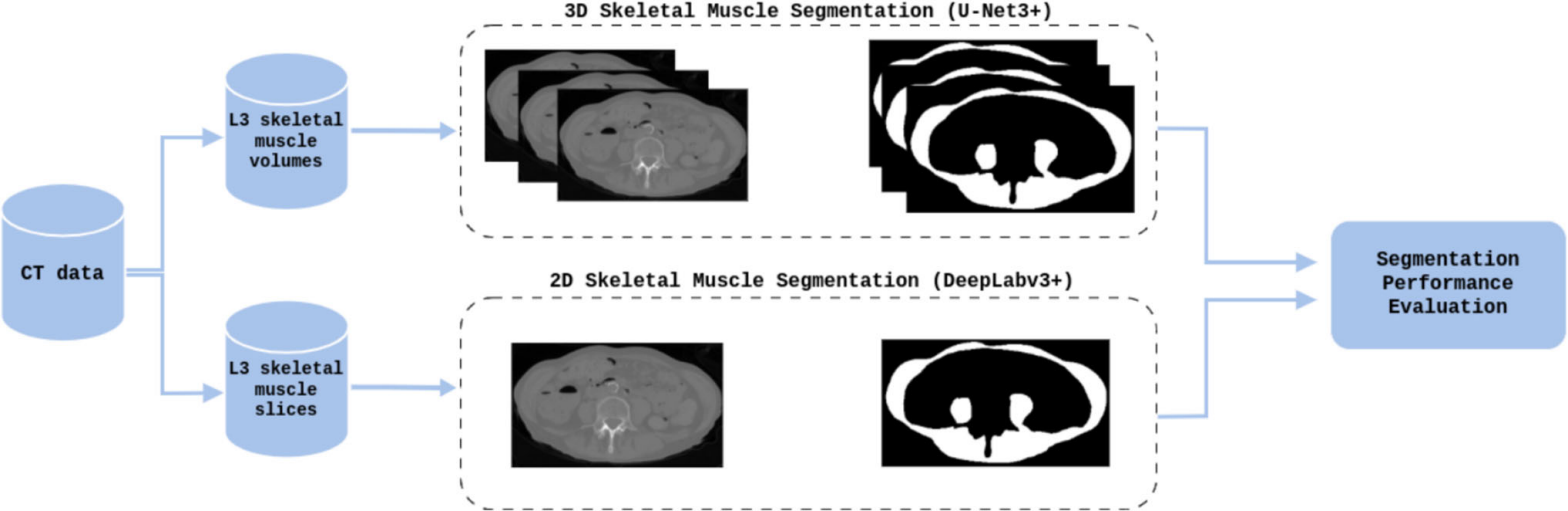
Proposed workflow for muscle segmentation. Starting from the same set of patients, two sub-datasets have been obtained (i.e., L3 skeletal muscle volumes and L3 skeletal muscle slices) for the implementation of volumetric (i.e., UNet3+) and two-dimensional (i.e., DeepLabv3+) segmentation models, respectively. Finally, the resulting segmentations were subsequently evaluated using metrics for evaluating the accuracy and robustness of segmentation models

Figure 1 illustrates the overall segmentation workflow. A dataset of 537 patients was used, from which we extracted: (i) three-dimensional subvolumes centered at the L3 level for training the UNet3+ model and (ii) single axial slices at the same anatomical level for training the DeepLabv3+ model. These subsets were preprocessed, augmented, and then split into training, validation, and test sets.

### Data Description

The CT scans used in this work are from a publicly available dataset described in [20], consisting of whole-body low-dose CT (LDCT) images acquired for PET/CT studies. The scans have an estimated effective dose between 1 and 2 mSv, with a reference dose of 200 mAs and tube voltage of 120 kV. The base segmentation masks were obtained from the open-access AtlasDataset [21], which provides anatomical labels for various musculoskeletal structures by aggregating annotations from multiple sources. No further manual refinement or exclusion criteria were applied; all labels were used as provided.

Each LDCT scan has a voxel resolution of approximately 0.90 $\times$ 0.90 $\times$ 2.5 mm. The original LDCT volumes are organized as three-dimensional matrices of size 512 $\times$ 512 $\times$ *N*, where $N = 460 \pm 55$ represents the number of axial slices per patient, depending on the individual anatomical coverage. While slice thickness and spacing on the *z*-axis are consistent across the dataset, in-plane resolution may vary slightly between patient scans.

The availability of full-body scans and the AtlasDataset annotation dataset allowed the extraction of consistent regions of interest centered on L3 region, making this dataset suitable for evaluating both volumetric and slice-based muscle segmentation approaches.

### Image Preprocessing and Data Preparation

The patients in the dataset were randomly divided into the following proportions: 80% (430 patients) for the training phase, 10% (54 patients) for the validation, and 10% (53 patients) for the test, aiming to guarantee a consistent training process between the two models and to ensure the comparability of their performances.

**Fig. 2:**
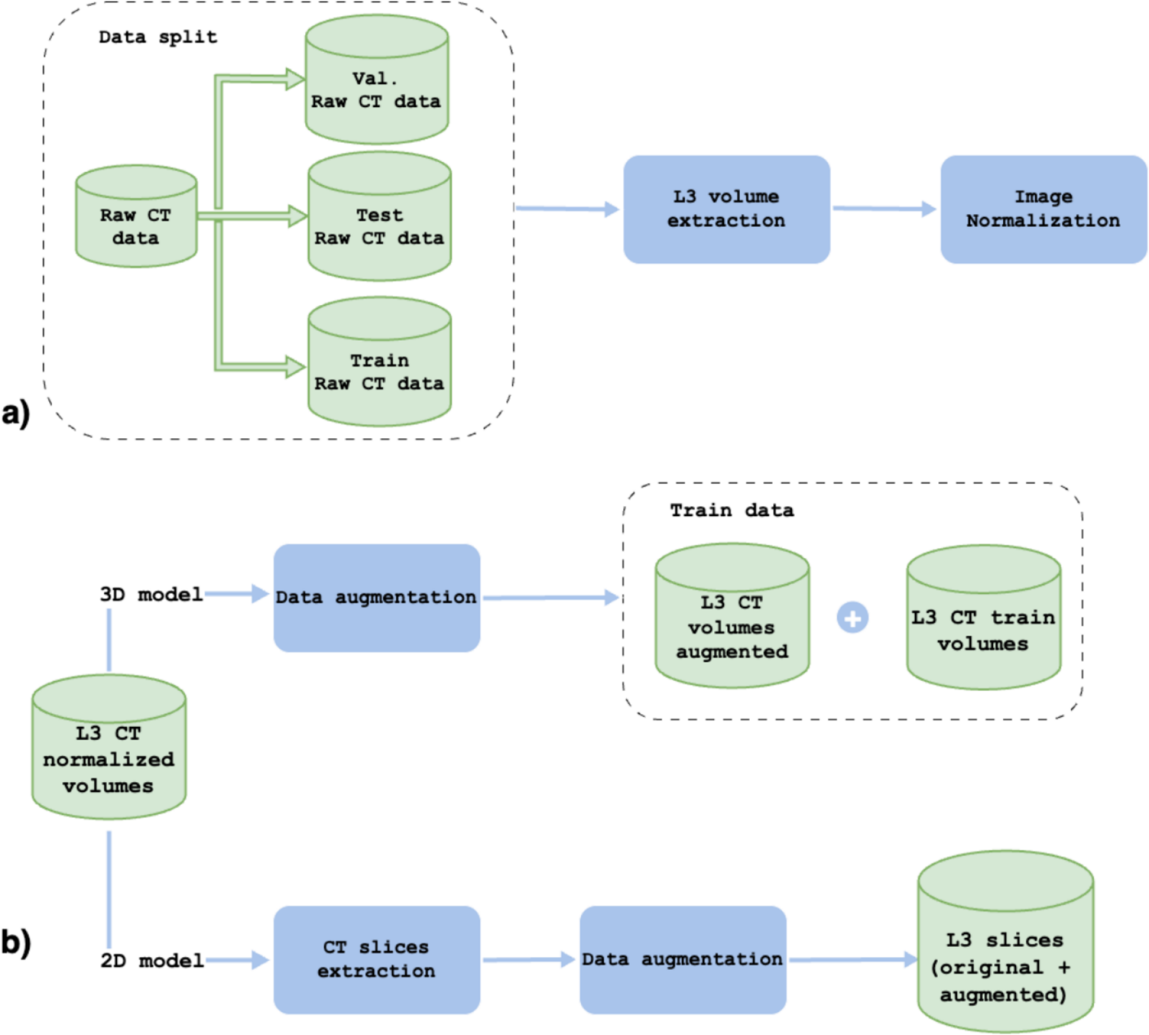
Data preprocessing pipeline for muscle segmentation: **a** raw CT scans are split into training, validation, and test sets; **b** axial L3 volumes are intensity-normalized and augmented for the 2D/3D segmentation models

A preprocessing pipeline was implemented to ensure consistency and reliability across the training, validation, and testing phases, as shown in Fig. 2.

As an initial step, HU thresholding was applied in the range within $-29$ and $+150$ to extract tissue regions with attenuation values characteristic of skeletal muscle. This allowed the exclusion of non-relevant tissues, such as visceral fat, which typically falls within a lower attenuation range between $-$190 and $-$29 HU [22]. Subsequently, regions of interest and corresponding muscle labels were extracted by selecting 20 consecutive axial slices at the level of the L3 vertebra. The number of slices extracted from the LDCT volume corresponds entirely to the maximum extractable volume of the manual annotations present in the dataset, which define the muscle volume of L3 approximately on 20 slices for each patient. This corresponds to a cranio-caudal extension of approximately 50 mm, based on an average slice thickness of 2.5 mm per scan.

Finally, a normalization step was performed on the LDCT volumes to standardize pixel intensity values. Specifically, a min-max normalization was applied using scaling voxel intensities to the range [0,1]. This step was necessary to prevent potential discrepancies and numerical instabilities during training, thereby enhancing model convergence and overall performance.

### Segmentation Network Architectures

In the context of skeletal muscle segmentation, UNet3+ and DeepLabv3+ architectures have been proposed for 3D and 2D segmentation, respectively.

For skeletal muscle volumetric segmentation, an adapted 3D version of UNet3+ architecture has been proposed as an evolution of UNet-based architectures.

UNet-based architectures are widely used in the medical field due to their symmetric encoder-decoder structure, which allows for combination of local and global information through direct connections between the corresponding encoder and decoder layers [23]. UNet-based models have demonstrated different semantic capacity between encoding and decoding features, reducing segmentation accuracy, especially for smaller anatomical structures. UNet++ mitigates this issue by using more complex connections between encoder and decoder, but at the cost of model complexity and redundancy [24] [25]. To improve the limitations of these previous versions, the UNet3+ architecture was introduced to enhance multi-scale feature fusion by concatenating decoder features with the corresponding encoder features at the same level as well as with those from higher encoder levels. This model allows for more comprehensive integration of semantic and spatial information across scales.

As in the original UNet architecture, skip connections are maintained between the encoder and decoder levels at the same scale. However, UNet3+ includes two additional types of skip connections to further improve the integration of multi-scale features. The first type refers to as intra encoder-decoder skip connections and it concatenates the features from higher-level encoder stages with those of lower-level decoder ones. This approach allows the decoder to access high-resolution features, although with lower semantic information, enriching the decoding process with spatial details and enhancing multi-scale features fusion. The second type of skip connection connects each decoder stage with all lower decoder levels enabling the integration of progressively refined features throughout the decoding hierarchy. Finally, the features obtained from these two additional types of skip connections are further concatenated in an unified representation improving the decoding process and segmentation performance [19]. These enhanced performances are reached by the UNet3+ architecture with fewer parameters than the previous version, contributing to a greater computational efficiency.

**Fig. 3:**
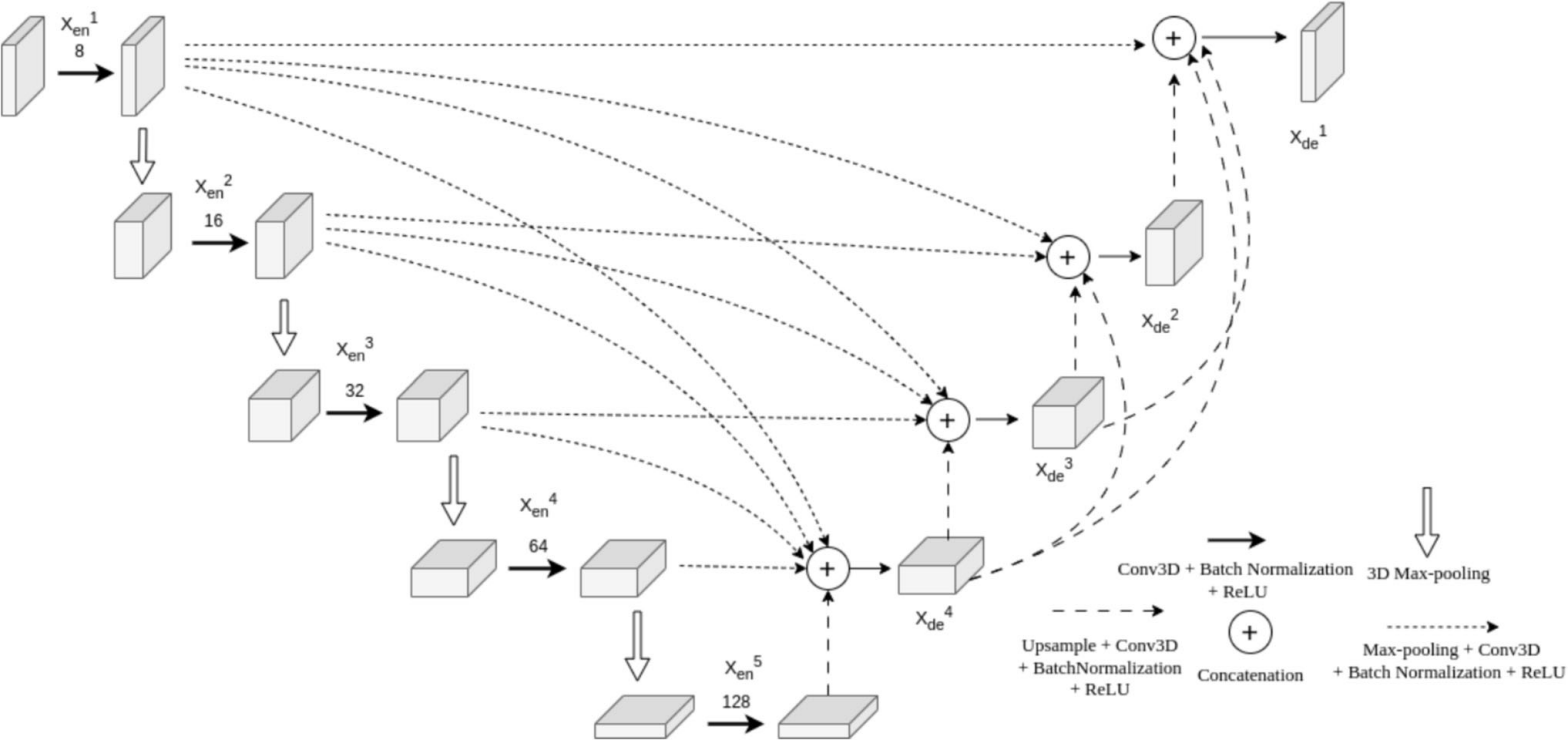
Schematic representation of the proposed 3D UNet3+ architecture. The model features a symmetric encoder-decoder structure with progressive feature channel expansion (8 to 128 filters). Multi-scale skip connections between encoder and decoder layers enable efficient spatial-semantic fusion while reducing the number of trainable parameters

Our UNet3+ proposed architecture is shown in Fig. 3. In the encoder path, the number of convolutional filters was progressively increased from 8 in the first layer to 128 in the bottleneck layer. This configuration was chosen to optimize GPU memory utilization while preserving adequate feature extraction capacity.

For 2D segmentation of muscle mass from single slices, the DeepLabv3+ architecture has been proposed. DeepLabv3+ is an extension of the DeepLabv3 model which acts as an encoder. A pre-trained convolutional neural network (e.g., ResNet, VGG16, VGG19) is used to extract both low-level and high-level features. Low-level features are extracted from the earlier layers and contain more spatial details, while high-level features are extracted from deeper layers and contain richer semantic information. Moreover, high-level features are further processed by the atrous spatial pyramid pooling (ASPP) module to capture multi-scale contextual information. These processed features are then fused with the low-level features in the decoder to refine spatial precision in the enhancing the final segmentation output [26].

**Fig. 4:**
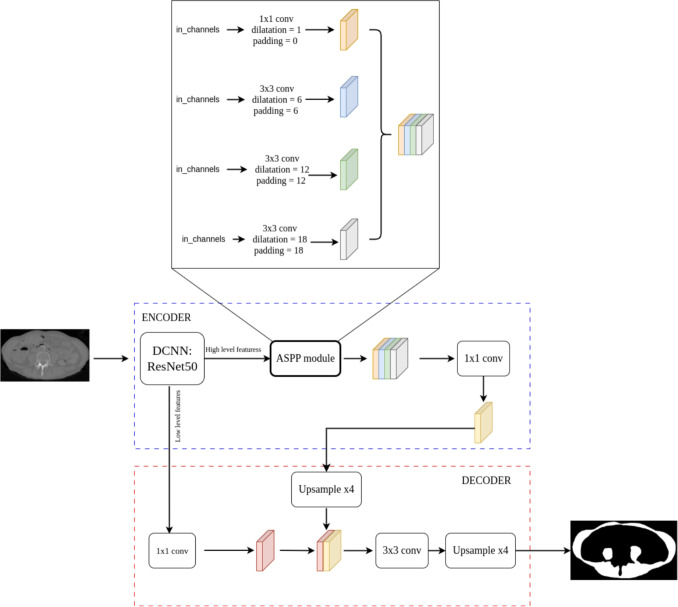
The proposed Deeplabv3+ architecture for 2D muscle segmentation. Architecture of the proposed DeepLabv3+ 2D model. The network uses a ResNet-50 backbone for multi-level feature extraction and integrates an ASPP (atrous spatial pyramid pooling) module from high-level features to acquire the multi-scale context. The decoder fuses the low-level features and those extracted by the ASPP module to ensure the quality of the final segmentation output

Our proposed DeepLabv3+ architecture is reported in Fig. 4. A pre-trained ResNet-50 backbone network was used to extract both high-level and low-level features. Moreover, the ASPP module was integrated into the encoder, and it employs a sequence of dilated convolutions with increasing dilatation rates, enlarging the receptive field of the high-level features and enhancing the model’s ability to capture contextual information across multiple spatial scales. High-level features processed by the ASPP module were concatenated with low-level features from the backbone network. This fusion is refined in the decoder path to produce the final segmentation output.

#### Model Training Setup

Each model was trained using a loss function suited to its design and segmentation strategy, as detailed below.

The slice-based 2D DeepLabv3+ model was trained using the Dice loss only, which is commonly used for slice-wise segmentation to maximize region overlap [27].

The volumetric 3D UNet3+ model was trained using a composite loss function combining Dice loss, focal loss, and structural similarity index measure (SSIM) loss. This hybrid formulation follows the original UNet3+ design [19] and was adapted to the 3D case to handle strong class imbalance, preserve fine boundaries, and enhance structural coherence across slices.

##### Dice Loss

This quantifies region overlap:

1 $$\begin{aligned} \mathcal {L}_{\text {Dice}} = 1 - \frac{2\sum _{1}^{N}p_{i}g_{i}}{\sum _{1}^{N}p_{i} + \sum _{1}^{N}g_{i}}. \end{aligned}$$

where $p_i$ represents the predicted probability of a pixel belonging to the target class, and $g_i$ is the corresponding ground truth label.

##### Focal Loss

This , a modification of binary cross-entropy (BCE) loss, emphasizes hard-to-classify voxels, mitigating class imbalance ([28] [29]):

2 $$\begin{aligned} \mathcal {L}_{\text {Focal}} = - \alpha _{t} (1 - p_{t})^{\gamma }\log (p_{t}). \end{aligned}$$

where $p_t \in (0,1)$ is the predicted probability for the true class, $\alpha _t \in [0,1]$ is the weighting factor for class balancing, and $\gamma \ge 0$ is the focusing parameter.

##### SSIM Loss

This measures structural similarity by combining luminance (4), contrast (5), and structure components (6) ([30] [31]):

3 $$\begin{aligned} \mathcal {L}_{\text {SSIM}}(x, y) = 1 - \left[ l(x, y) \right] ^\alpha \cdot \left[ c(x, y) \right] ^\beta \cdot \left[ s(x, y) \right] ^\gamma , \end{aligned}$$

4 $$\begin{aligned} l(x, y) = \frac{2\mu _x \mu _y + C_1}{\mu _x^2 + \mu _y^2 + C_1} \end{aligned}$$

5 $$\begin{aligned} c(x, y) = \frac{2\sigma _x \sigma _y + C_2}{\sigma _x^2 + \sigma _y^2 + C_2} \end{aligned}$$

6 $$\begin{aligned} s(x, y) = \frac{\sigma _{xy} + C_3}{\sigma _x \sigma _y + C_3} \end{aligned}$$

where $\mu _x$ and $\mu _y$ denote the mean intensity values of images *x* and *y*, respectively, while $\sigma _x$ and $\sigma _y$ are their standard deviations. The term $\sigma _{xy}$ represents the covariance between the two images. The constants $C_1$, $C_2$, and $C_3$ are introduced to stabilize the division when denominators are close to zero. The exponents $\alpha$, $\beta$, and $\gamma$ are weighting factors.

The final loss for UNet3+ is then as follows:

7 $$\begin{aligned} \mathcal {L}_{\text {UNet3+}} = \mathcal {L}_{\text {Dice}} + \mathcal {L}_{\text {Focal}} + \mathcal {L}_{\text {SSIM}}. \end{aligned}$$

This configuration ensures that each model leverages an appropriate optimization strategy aligned with its architecture and the demands of the segmentation task.

**Fig. 5:**
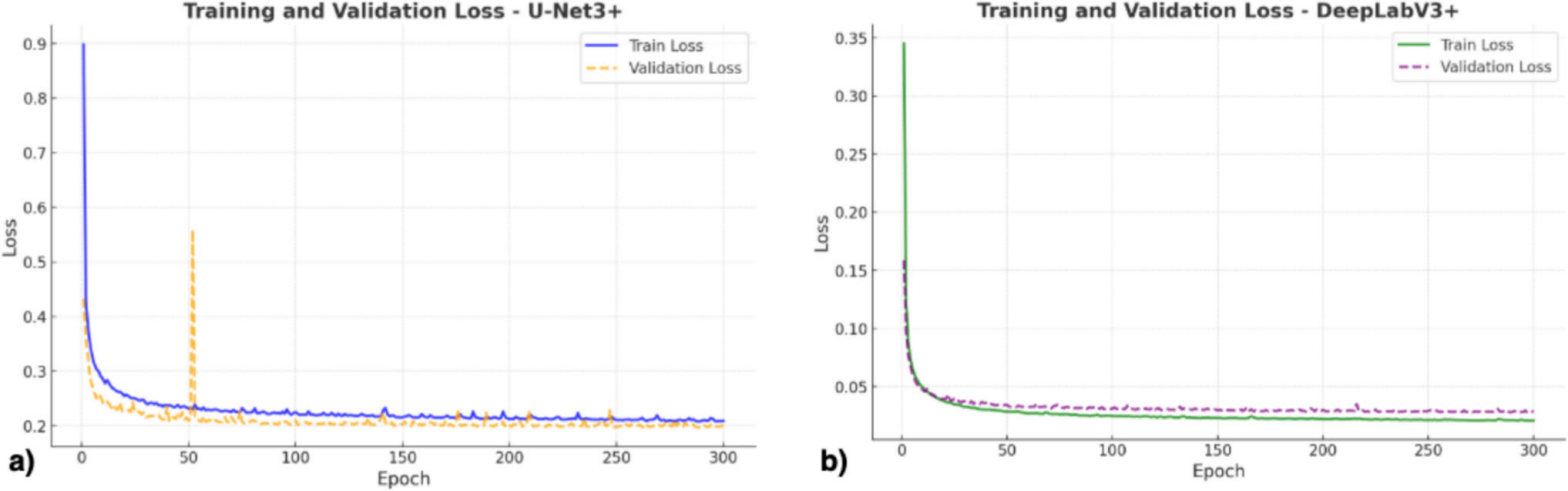
Loss function trend for training and validation in UNet3+ (**a**) and DeepLabV3+ (**b**) models. The trend of the loss curves obtained during the training phase of the models shows the absence of overfitting

## Results

The proposed 3D and 2D networks were trained and tested using LDCT scans of a cohort of 537 patients. From this dataset, 430 patients were used for model training, and 54 patients were used for model validation during training, while model performance testing was performed on an extracted subset of 53 patients.

The pipeline implementing the two proposed models was written in Python 3.8.10 using PyTorch [32] and MONAI libraries [33].

Both the UNet3+ and DeepLabv3+ models were trained for 300 epochs using a learning rate of $10^{-4}$, and their performance during training was monitored using the Dice loss score computed on the validation dataset in order to assess each model’s generalization capability.

The UNet3+ model was trained using eight NVIDIA RTX 2080 Ti GPUs through model parallelism, whereby the network layers were distributed across the available GPUs. This strategy allowed the modular structure of the UNet3+, enabling effective load balancing and optimizing the use of the available computational resources for tensor computation. The training was carried out on a server equipped with an Intel Xeon Gold 5118 processor and 256 GB of RAM.

The DeepLabv3+ model was trained on the same hardware platform but using a single NVIDIA RTX 2080 Ti GPU. Since the input data were 2D and each batch required relatively low computational resources compared to the 3D model, multi-GPU parallelization was not necessary.

Training loss curves for both models (Fig. 5) confirm stable learning and absence of overfitting.

Resulting skeletal muscle segmentations at L3 from both models were evaluated from a volumetric perspective to allow a direct comparison between the two approaches. The performance of the proposed segmentation models was evaluated using multiple quantitative metrics to ensure a comprehensive assessment of their accuracy and reliability. In accordance with the guideline proposed by Müller et al. [34], performance was evaluated using multiple complementary metrics, (i) including overlap (DSC), (ii) boundary (HD95, ASD), and (iii) pixel-wise metrics (sensitivity, specificity, precision), in order to capture complementary aspects of accuracy.

**Table 1:** Performance metrics and model complexity on the test dataset for the proposed architectures

Method	DSC	HD95	ASD	Sens.	Spec.	Prec.	Params
		[mm]	[mm]				
UNet3+	0.967	1.28	0.33	0.970	0.997	0.965	1.27M
	±0.013	±0.59	±0.28	±0.009	±0.001	±0.0451	
DeepLabv3+	0.980	1.04	0.19	0.980	0.998	0.973	33.6M
	±0.010	±0.46	±0.12	±0.021	±0.001	±0.009	

*DSC* Dice similarity coefficient, *HD*95 95th percentile Hausdorff distance, *ASD* average surface distance, *Sens*. sensitivity, *Spec*. specificity, *Prec*. precision

The Dice similarity coefficient (DSC) was considered to quantify the spatial overlap between the predicted segmentation and the ground truth, providing an overall measure of segmentation performance [34]. It ranges from 0 (for non-coincident segmentation maps) to 1 (for perfectly coincident segmentation maps).

As a complementary metric to the overlap metrics, we compute the 95th percentile Hausdorff distance (HD95), which captures the amount of deviation at the boundary between the predicted segmentation and the ground truth. Unlike the maximum Hausdorff distance, HD95 is more robust to outliers, reporting the worst-case error for 95% of the surface points. It is expressed in millimeters, with values close to 0 mm indicating a perfect match between the two segmentation maps. [35].

The average surface distance (ASD) is used to calculate the average point-to-point difference of the predicted segmentation from the ground truth along the surface of the segmented object [36]. While HD95 identifies the worst errors, ASD provides a more reliable measure of global edge consistency, capturing the overall quality of the segmented surface. It is expressed in millimeters as a non-negative real number, and an ideal alignment would be 0 mm [35].

For pixel-by-pixel classification, sensitivity, also known as recall or true positive rate, focuses on the model’s ability to detect true positives, while specificity, also called true negative rate, assesses the model’s ability to identify true negatives [34]. Thus, in this context, a high sensitivity ensures the correct identification of muscle tissue, minimizing false negative pixels, while a high specificity ensures an excellent ability of the model to exclude non-muscle regions.

The performance metrics of both models are reported in Table 1, together with the number of trainable parameters. DeepLabv3+ achieved a higher average Dice similarity (0.980 ± 0.010) than UNet3+ (0.967 ± 0.013) and slightly lower boundary errors in both HD95 and ASD. The 2D model also yielded higher sensitivity, specificity, and precision, albeit with small absolute differences.

However, UNet3+ reached competitive segmentation performance while using only 1.27 million parameters, compared to 33.6 million for DeepLabv3+. This corresponds to over a 26-fold reduction in model complexity, highlighting the efficiency of the 3D architecture.

**Table 2:** Inference time comparison between UNet3+ and DeepLabv3+ models on the test dataset

Model	Inference time (s)
UNet3+ (only forward pass)	0.0161 ± 0.0447
UNet3+ (after de-resize)	0.2116 ± 0.0200
DeepLabv3+	0.2966 ± 0.0361

Values are reported as mean ± standard deviation in seconds

$^{*}$ “Only forward pass” refers to the raw model inference without post-processing

$$^{**}$$ “After de-resize” includes the time required to restore original image dimensions after prediction

Table 2 reports the inference time per patient for both models. UNet3+ completed volumetric segmentation in 0.0161 s on average, significantly faster than DeepLabv3+, which required 0.2966 s per patient. This difference was statistically significant ($p < 0.00001$) with an exceptionally large effect size (Cohen’s $d = 7.224$).

To further assess the performance differences, a paired *t*-test and effect size analysis were performed (Table 3). Statistically significant improvements were observed for DeepLabv3+ in Dice similarity ($p = 0.00026$, $d = 0.538$), recall ($p = 0.00005$, $d = 0.605$), precision ($p = 0.0229$, $d = 0.322$), and specificity ($p = 0.0255$, $d = 0.316$). In contrast, no significant differences were found in HD95 ($p = 0.537$) or ASD ($p = 0.767$), indicating comparable accuracy in boundary localization between the two models.

**Table 3:** Statistical comparison between DeepLabv3+ (2D) and UNet3+ (3D) on the test dataset

Metric	*p*-value (paired *t*-test)	Cohen’s *d*
DSC	**0**.**00026**	0.538
HD95 (mm)	0.537	$-$0.085
ASD (mm)	0.767	$-$0.041
Precision	**0**.**0229**	0.322
Recall (sensitivity)	**0**.**00005**	0.605
Specificity	**0**.**0255**	0.316
Inference time (s)	**<0.00001**	7.224
(only forward pass)		
Inference time (s)	**<0.00001**	4.543
(after de-resize)		

Paired *t*-test, *p*-values, and Cohen’s *d* effect sizes are reported for each metric

Bold *p*-values indicate statistically significant differences ($p < 0.05$). Cohen’s *d*: 0.2 = small, 0.5 = medium, 0.8+ = large effect size

$^{*}$ “Only forward pass” refers to the raw model inference without post-processing

$$^{**}$$ “After de-resize” includes the time required to restore original image dimensions after prediction

**Fig. 6:**
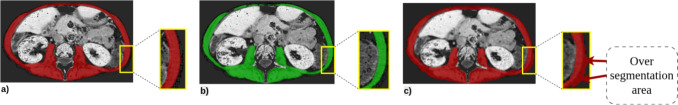
Qualitative comparison of worst-case segmentation performance for UNet3+ (DSC = 0.8279) and DeepLabv3+ (DSC = 0.9150). **a** DeepLabv3+ prediction (2D), **b** ground truth segmentation, and **c** UNet3+ prediction (3D). The red arrows highlight an over-segmentation area in the UNet3+ prediction, involving the lateral border of the psoas muscle. As shown, the DeepLabv3+ 2D prediction preserved a more anatomically accurate and conservative muscle contour. In this example, other muscle regions are also over-segmented by the UNet3+ model

## Discussion

The main contribution of this study is the comparative evaluation of two deep learning architectures—UNet3+ and DeepLabv3+—for site-specific segmentation of skeletal muscle at the L3 vertebral level on LDCT images. To the best of our knowledge, this is the first work that systematically evaluates and compares 2D and 3D segmentation architectures in this clinical context, also introducing UNet3+ for volumetric segmentation of skeletal muscle.

UNet3+ demonstrated good segmentation capabilities (DSC=0.967) despite having only 1.27 million parameters—over 26 times less than DeepLabv3+—and operating on full 3D volumes. DeepLabv3+ achieved superior performance in most metrics (DSC = 0.980), in particular in terms of sensitivity and recall, confirming its effectiveness in acquiring truly positive muscle regions.

The observed performance differences are attributable to several factors. First, DeepLabv3+ operates on full-resolution 2D slices, preserving finer spatial details and allowing for more precise localization of muscle boundaries. In contrast, the 3D model required spatial downsampling during training to accommodate memory constraints, which may have led to the loss of anatomical details, especially along complex or low-contrast boundaries. Additionally, the 2D model can benefit from architectural features such as atrous spatial pyramid pooling (ASPP) which is essential for capturing multi-scale context in planar images.

With 430 training patients, our dataset may have been insufficient for the 3D model to fully exploit volumetric relationships. Three-dimensional networks typically require larger datasets to learn complex spatial relationships across slices effectively, especially when larger slice thickness makes this more challenging. The observed performance gap might diminish with larger training cohorts, suggesting that our results reflect data availability constraints rather than fundamental architectural limitations.

In fact, in anatomically complex cases where the 2D and 3D models performed the worst in the test set, the 3D model tended to oversegment peripheral soft tissue regions, likely due inter-slice connectivity that introduced smoothing artifacts in anatomically complex regions. As illustrated in Fig.  6, the 2D model produced more anatomically accurate segmentations, aligning better with finer boundaries. These results suggest that while volumetric models provide context-based predictions, they may require further refinement in more complex anatomical cases, such as those with low contrast or structural variability.

Despite these differences, UNet3+ demonstrated robust and consistent segmentation performance, with comparable boundary accuracy and a significant advantage in computational efficiency, making it suitable for scalable or real-time clinical applications. This advantage is confirmed by the inference time analysis reported in Table 2, where UNet3+ achieved a mean forward-pass time of only 0.016 s per scan. Even when including the post-processing step required to restore the original image resolution (de-resizing), the total processing time remained well below that of DeepLabv3+ (0.211 s vs. 0.297 s). This makes UNet3+ not only computationally lightweight but also efficient for deployment in time-sensitive workflows, such as real-time assessment or large-scale screening programs.

In comparison with previous works that used standard-dose CT (Table 4), the proposed models achieved competitive segmentation performance, confirming the feasibility of using LDCT for quantitative muscle analysis. Additionally, unlike most prior studies, our evaluation included boundary metrics (HD95, ASD) and pixel-wise classification scores (sensitivity, specificity), offering a more robust and multifaceted understanding of model behavior.

However, our study has several limitations. The dataset was limited to 537 annotated scans from a single source, and no external validation was performed. This reduces the generalizability of the results to different clinical settings, scanners, or patient populations. Performance metrics from one annotation source cannot predict behavior across different imaging protocols, scanner manufacturers, or institutional annotation standards. The absence of external validation renders our performance claims institution-specific rather than broadly applicable.

**Table 4:** Comparison of the proposed segmentation models with literature approaches

Authors, year	Dataset	DL Model	DSC	Sens	Spec	HD95 (mm)
Nowak et al., 2022 [37]	240 CT scans	CDFNet	0.950 ± 0.040	n.a	n.a	n.a
Ackermans et al., 2021 [38]	3413 CT scans	2D UNet	0.926 (IQR: 0.866–0.959)	n.a	n.a	n.a
Park et al., 2020 [39]	426 CT scans	FCN-based	0.970	n.a	n.a	n.a
Dabiri et al., 2020 [40]	3774/1801 slices	UNet-based	0.975	0.970	0.990	n.a
He et al., 2023 [41]	262 CT scans	2D nn-UNet	0.968	n.a	n.a	n.a
Magudia et al., 2021 [42]	604 patients	2D UNet	0.960	n.a	n.a	n.a
Castiglione et al., 2021 [43]	370 patients	2D UNet	0.930	n.a	n.a	n.a
Roblot et al., 2022 [13]	1025 slices	DeepLabv3	0.910	0.957 ± 4.000	n.a	6.90 ± 4.00
**This work (3D)**	430 LDCT scans	**UNet3+**	**0.967 ± 0.013**	**0.967 ± 0.009**	**0.997 ± 0.001**	**1.27 ± 0.58**
**This work (2D)**	430 LDCT scans	**DeepLabv3+**	**0.982 ± 0.010**	**0.982 ± 0.011**	**0.998 ± 0.001**	**1.04 ± 0.46**

Metrics are reported as mean ± standard deviation where available. *n*.*a*. not available

**Note:** **Many studies in the literature do not report boundary-level accuracy metrics (HD95, ASD), limiting their evaluation to DSC or overlap-based measurements. For such works, these values are reported as n.a. In contrast, this study reports both overlap-based and boundary-level metrics, providing a more comprehensive performance evaluation**

Furthermore, since patient height was not available, it was not possible to calculate clinically meaningful indices such as the skeletal muscle index (SMI) to assess the applicability in the clinical setting to assess for example some clinical conditions such as sarcopenia. Therefore, the study remains focused on the performance of the technical segmentation, without a direct evaluation of the utility of the models in sarcopenia detection.

Furthermore, although this study represents a first comparison between 2D and 3D segmentation approaches on LDCT images, further analysis involving other state-of-the-art architectures present in the literature will be needed in order to more accurately assess the performance variability and generalizability of the models. The aim of this work was primarily to explore the feasibility of using volumetric models such as UNet3+ for muscle segmentation in low-dose settings, while being aware that performance could be further improved through alternative training configurations or strategies.

Despite these limitations, the results demonstrate the technical feasibility of automated muscle segmentation on LDCT images and provide the first systematic comparison of 2D vs 3D approaches in this context. The computational efficiency of UNet3+ particularly highlights the potential for volumetric approaches in clinical workflows, while the superior accuracy of DeepLabv3+ confirms the continued relevance of 2D methods for high-precision segmentation applications on LDCT images.

## Conclusion

This study presented a comparative evaluation of two deep learning models—UNet3+ (3D) and DeepLabv3+ (2D)—for segmenting skeletal muscle at the L3 vertebral level on low-dose CT scans. While DeepLabv3+ achieved slightly higher accuracy in terms of overlap and pixel-by-pixel metrics, UNet3+ demonstrated strong boundary consistency and remarkable computational efficiency, making it a promising option for real-time or large-scale screening applications.

The results confirm that reliable skeletal muscle segmentation can be achieved even in low-dose imaging settings, supporting the clinical feasibility of such approaches. Furthermore, volumetric models such as UNet3+ provide a more complete anatomical representation than slice-based methods, which may increase their utility in assessing body composition and conditions such as sarcopenia.

Future work should aim to validate these models on external datasets, incorporate clinically meaningful endpoints such as the skeletal muscle index (SMI), and explore additional architectures to further optimize performance in anatomically challenging cases. Additionally, transfer learning strategies could be investigated, whereby the 3D segmentation model is initialized using weights from a well-performing 2D model. This approach may enhance convergence speed and generalization, particularly in scenarios with limited training data.

### Supplementary Information

No supplementary material is available for this study.

## Data Availability

The dataset used in this study is available at TCIA under the title FDG-PET-CT-Lesions and was accessed upon formal request (10.7937/gkr0-xv29).

## Code Availability

The full code implementation and the pre-trained model weights for both UNet3+ and DeepLabv3+ are available at https://github.com/GiuseppeTimpano/Muscle_segmentation.
